# Advancements of physical exercise and intestinal microbiota and their potential mechanisms

**DOI:** 10.3389/fmicb.2025.1595118

**Published:** 2025-07-23

**Authors:** Rendong Li, Tianning Zhang, Zhen Cheng, Chenxi Yao, Xiangguang Meng, Tie Ma

**Affiliations:** ^1^Department of Physical Education, Shenyang University of Chemical Technology, Shenyang, China; ^2^Northeastern University, College of Life and Health Sciences, Shenyang Sport University, Shenyang, China; ^3^College of Food Science, Shenyang Agricultural University, Shenyang, China; ^4^Graduate School of Beijing Sport University, Beijing Sport University, Beijing, China; ^5^Yingkou Vocational and Technical College, Yingkou, China; ^6^College of Exercise and Health, Shenyang Sport University, Shenyang, China

**Keywords:** exercise, gut microbiota, body functions, exercise capacity, skeletal muscle anabolism

## Abstract

Gut microbiota is closely related to body functions. At present, evidence has shown that gut microbiota and its metabolites interact with exercise, but the effects and related mechanisms are still in the exploratory stage. Therefore, this paper summarizes the results of experiments related to exercise and gut microbiota, and analyzes the possible mechanism of the interaction between the two, in order to provide a theoretical basis for further research on the relationship between exercise and gut microbiota. The intervention of gut microbiota may be an effective help to improve exercise capacity. The abundance, composition and diversity of gut microbiota can affect the body’s exercise quality. We conclude, the relationship between exercise and gut microbiota is closely related and not only determined by a single influencing mechanism, which provides a new perspective and direction for future research on the relationship between exercise and gut microbiota.

## 1 Introduction

All kinds of microorganisms in the intestinal tract of the body are collectively referred to as intestinal microbiota. Human gut microbiota contains thousands of different bacteria, as well as various archaea, eukaryotic microorganisms and viruses (Pitocco et al., 2020), which have great metabolic capacity (Mailing et al., 2019). The gut microbiota is mainly composed of *Firmicutes, Bacteroidetes, Proteobacteria,* and *Actinobacteria*, of which *Firmicutes* and *Bacteroidetes* are the most abundant, accounting for 90% of the human gut microbiota (Hu et al., 2022; Banaszak et al., 2023). About one third of the gut microbiota is common to most people, while the other two thirds are unique to individuals (Campbell and Wisniewski, 2017). Gut microbiota and the host coevolve, forming a complex interweave and mutualism relationship (Clauss et al., 2021).

There is an interactive relationship between the body and gut microbiota. The composition of the body’s gut microbiota can be affected by many factors, including age, diet, drugs, and delivery mode (Mailing et al., 2019; Chernikova et al., 2019). Studies on multiple populations have found that exercise can also affect the diversity and composition of gut microbiota, but there are some contradictions and differences in the effects of exercise on gut microbiota of different populations, which need further sorting and exploration (Clauss et al., 2021). Gut microbiota can also have an impact on body function. Gut microbiota can maintain the integrity and permeability of the intestine (Di Tommaso et al., 2021; Di Vincenzo et al., 2024), promote the host to obtain nutrients and energy from food, and synthesize and release a large number of metabolites, such as short-chain fatty acids and amino acids (Puljiz et al., 2023; Zhang et al., 2023). These metabolites also have rich physiological functions, and they can enter the host metabolic circulation and exert their effects outside the intestine (Mailing et al., 2019; Makin, 2021). Therefore, gut microbiota can have effects on local intestinal and systemic organs. For example, studies have shown that gut microbiota can affect the function of brain and internal organs (Mailing et al., 2019; de Vos et al., 2022), and the species and abundance of gut microbiota are related to the occurrence and development of a variety of diseases (Nie et al., 2024; Kim et al., 2024). However, there are few studies on the effect of gut microbiota on exercise capacity.

Based on the existing research results on the relationship between gut microbiota and various organs, cells and molecules in the body, this study provides a theoretical basis for the relationship between exercise and gut microbiota. Studies have found that exercise may lead to changes in the composition of gut microbiota by reducing obesity, enhancing intestinal metabolic activity, and participating in the regulation of hypothalamic–pituitary–adrenal axis and gut-brain axis (Wegierska et al., 2022). The immune system is a complex cellular network that is widely distributed in the human body and plays a key role in many functional processes (Sender et al., 2023). Exercise affects the production of some metabolites in the body, such as oxylipin, plasma TCA metabolites, immune-related proteins, etc., which can participate in the regulation of immune response (Nieman and Pence, 2020). At the same time, some molecules in the innate immune system and the acquired immune system, including antimicrobial peptides and immunoglobulin A, can participate in the regulation of the homeostasis and diversity of gut microbiota (Zheng et al., 2020). The gut is the organ containing the most immune cells in the human body, and relies on the immune system to communicate with the brain and participate in the signal transduction of the gut-brain axis (Asadi et al., 2022). Studies have shown that repetitive aerobic exercise can improve brain structure and function. When the exercise intensity exceeds 60% of the maximal oxygen uptake of the individual or the exercise time exceeds 90 min, the activation of gut-brain axis and the change of gut microbiota homeostasis will occur (Dalton et al., 2019). The hypothalamic–pituitary–adrenal axis plays a central role in the regulation of the gut-brain axis (Rusch et al., 2023). The hypothalamic–pituitary–adrenal axis can be activated due to environmental stress or increased proinflammatory cytokines, and its secretion of cortisol can change intestinal permeability and the composition and diversity of gut microbiota (Jang et al., 2020). The connections between various parts of the body are complex and diverse, and the relationship between the microbiota and the rest of the body is bidirectional and symbiotic (Álvarez-Herms and Odriozola, 2024). Not only can exercise cause changes in gut microbiota by affecting body functions, but also when the homeostasis of gut microbiota changes, the influence of its homeostasis on a variety of body functions will also be reflected in changes in exercise capacity (Fritz et al., 2024; Ni Lochlainn et al., 2024; Chen Y. et al., 2024). Exercise and gut microbiota may interact with each other through a variety of ways, but the specific mechanisms need to be further studied.

Although evidence has shown the potential association between exercise and gut microbiota, the effects of exercise on gut microbiota vary greatly in different populations, and the mechanisms underlying the interaction between exercise and gut microbiota are still in the exploratory stage. Therefore, this article will review the interaction between exercise and gut microbiota and its mechanism, so as to provide basis and direction for further research on the relationship between exercise and gut microbiota.

## 2 Effects of exercise on gut microbiota

The influence of exercise on body function is significant and multifaceted. It is generally believed that the stimulation of the body to exercise is mainly manifested in the adaptability of long-term regular training and the stress of short-term one-time exercise. Therefore, the study on the effects of exercise on gut microbiota and metabolites will be carried out from two aspects: long-term exercise and one-time exercise.

### 2.1 Effects of long-term exercise on gut microbiota and its metabolites

Athletes are a group of people who have been engaged in systematic training for a long time. Taking rugby players who have been trained for a long time as the research object, it is found by sequencing the 16S rRNA gene that the diversity of gut microbiota of rugby players of the same sex under the condition of uncontrolled diet is higher than that of non-athletes of similar age and BMI. And the higher the level of rugby players, the higher the relative expression of *Ruminococcus*, *Succinivibrionaceae*, *Succinivibrio*, *Prevost* and *Akkermansiaceae* in their gut microbiota (Campbell and Wisniewski, 2017). In a cross-sectional comparison of cyclists with different amounts of exercise, cyclists who exercised more than 16 h per week had a higher abundance of *Prevotella* (Petersen et al., 2017). Exercise intervention can change the abundance of *Methanobrevibacter* in the intestine of cyclists, and it is believed that methane metabolism will increase with the increase of the abundance of *Methanobrevibacter*, followed by the up-regulation of carbohydrate and three kinds of energy metabolism, thus having a wider impact on the body (Petersen et al., 2017). Compared with ordinary elderly people, elderly athletes have more beneficial bacteria and fewer harmful bacteria in their intestines, which means that long-term exercise training may help to resist gastrointestinal health damage caused by aging (Fart et al., 2020). However, some scholars believe that for athletes, long-term endurance exercise has a broader negative impact on their gut microbiota, which is manifested as reducing the diversity of gut microbiota and increasing intestinal permeability (Bonomini-Gnutzmann et al., 2022).

For non-athletes, 17 overweight or obese postmenopausal women were taken as the research object, and after 12 weeks of intervention with high-intensity intermittent exercise combined with resistance exercise, it was found that their abdominal and visceral fat mass decreased, segmental muscle mass increased, and the composition of gut microbiota of the subjects also changed. Some gut microbiota are related to the change of body composition, for example, the abundance of *Bifidobacterium* is positively related to fat quality and negatively related to muscle mass; However, *Prevotella* is negatively correlated with fat quality and positively correlated with muscle mass (Dupuit et al., 2022). Therefore, the author thinks that the improvement of body composition by exercise may be mediated by gut microbiota. In addition, studies have found that exercise can also affect the gut microbiota of non-obese subjects, but the characteristics of the changes are different with the degree of obesity. For example, after 6 weeks of exercise intervention, sedentary adults with different body types are given 30–60 min each time three times a week, it is found that the gut microbiota of thin people is significantly increased by *Faecalibacterium* spp. and *Lachnospira* spp., while that of *Collinsella* spp. is slightly decreased after exercise intervention; However, the indexes of *Collinsella* spp. and *Lachnospira* spp. in obese people have increased, and the indexes of fecal bacteria have decreased significantly (Allen et al., 2018). In addition, the patients with metabolic syndrome were intervened by Wuqinxi exercise [Wuqinxi, also called gymnastics of 5 animals in foreign countries, is a traditional form of exercise comes from the ancient Daoyin, it has been used to prevent disease, cure diseases and health care for a long time (Zhang et al., 2018)] for 6 months, 6 days a week and 40–50 min a day. The results showed that the intestinal microecology of the host was optimized, and the *Bifidobacterium*, *Lactobacillus*, *Bacteroides* and *Clostridium* in the patients increased, while the *Fusobacterium*, *Enterococcus*, *Staphylococcus* and *Veillonella* decreased. The longer the intervention time, the more obvious the effect. At the same time, this study also showed that the intervention of Wuqinxi exercise could effectively improve the symptoms of patients. Therefore, the authors believe that long-term Wuqinxi exercise can improve the structure of the host’s gut microbiota and alleviate the patient’s metabolic syndrome (Sun and Zhong, 2019). In addition, different forms of exercise were found to have different effects on gut microbiota. For example, an 8-week endurance and resistance training program was conducted in young women, and it was found that strength training did not cause significant changes in gut microbiota. Endurance training to improve cardiopulmonary fitness has a significant effect on the gut microbiota of subjects, although this effect only appears in the early stage of exercise (Bycura et al., 2021). Animal experimental studies have also shown that endurance or resistance training can change the gut microbiota of mice at the genus and species levels, but compared with strength training, endurance training can make the gut microbiota of mice have higher diversity and uniformity (Fernández et al., 2021). However, another study found that 26 middle-aged patients with insulin resistance were given a two-week, three times a week exercise intervention, which included intermittent sprint exercise and moderate-intensity endurance exercise. It was found that the changes of gut microbiota of the subjects were similar between the different exercise modes, showing the increase of *Bacteroidetes* and the decrease of *Firmicutes/Bacteroidetes* ratio (Motiani et al., 2020). The reason for the difference may be related to the similar disease status of the subjects and the short exercise time. Long-term exercise intervention will cause changes in the diversity and composition of gut microbiota in different groups. However, it can be seen that different groups have different types of gut microbiota changes, which may be related to the different ways of exercise or the different basic gut microbiota states of the studied groups. In addition, most studies did not control diet, which has a direct and important effect on gut microbiota. Therefore, at this stage, the effect of long-term exercise on gut microbiota needs further research, which can be carried out by refining the exercise program, targeting specific groups, limiting or investigating the diet.

In addition to affecting the composition of gut microbiota, exercise also affects the expression of its metabolites. Gut microbiota can produce a variety of metabolites, such as bile acids, short-chain fatty acids, amino acids, choline and ethanol (Chen and Vitetta, 2020). At present, most of the research focuses on the metabolites of short-chain fatty acids (SCFA) and amino acids during exercise. Studies have found that athletes have higher levels of SCFA than sedentary people (Mohr et al., 2020). The SCFA of non-athletes is also affected by physical activity, and when they increase their physical activity, the relative abundance of SCFA increases (Dziewiecka et al., 2022). In the absence of changes in dietary patterns, 6-week exercise intervention tended to increase the fecal SCFA in sedentary groups. In contrast, the increase of SCFA in sedentary obese groups was small and lasted for a short time (Allen et al., 2018). In addition, long-term exercise intervention in patients with metabolic syndrome has also been found to improve the number of SCFA-producing bacteria in the subjects (Sun and Zhong, 2019). Animal studies have also shown that the SCFA synthesis of gut microbiota of diabetic mice increased after swimming exercise intervention for 5 days a week for more than 8 weeks (Valder and Brinkmann, 2022). Therefore, it can be seen that compared with the complexity of gut microbiota after long-term training adaptation, the changes of SCFA in response to exercise are relatively consistent.

### 2.2 Effects of one-time exercise on gut microbiota and its metabolites

At present, the effect of one-time exercise on gut microbiota is mostly concentrated in athletes. For example, studies have found that after participating in a marathon, the abundance of *Veillonella* in the feces of athletes increases (Makin, 2021). Four male athletes participated in a 33 day and 5,000 km ocean rowing race, and it was found that the *α*-diversity and the overall relative abundance of some microorganisms in the gut microbiota of the athletes were increased. The gut microbiota of these four athletes showed an increase in the abundance of butyric acid-producing microbiota related to improving insulin sensitivity, and intestinal microbial genes involved in the biosynthesis of specific amino acids and fatty acids (such as *B. vulgatus* and *F. prausnitzii*). The expression of *Coprococcus* spp. *art 55/1*, *Enterobacter* (*R. intestinalis*), Bacteroides vulgaris (*B. vulgatus*), *Monobacteroides* (*B. uniformis*) involved in l-isoleucine metabolic pathway also increased (Keohane et al., 2019). Although there was no significant change in the composition diversity of gut microbiota, the abundance of some gut microbiota and amino acid metabolites (such as tryptophan, tyrosine, and phenylalanine) were increased in male cross-country non-professional long-distance runners undergoing a single moderate-intensity exhaustive exercise. Metabolomics analysis found that some metabolites in serum came from gut microbiota. Therefore, the authors speculated that the changes of metabolites in some serum may be related to the changes of gut microbiota induced by exercise (Tabone et al., 2021). In conclusion, one-time exercise can have a significant effect on gut microbiota, this effect is different, which may be due to the fact that most of the subjects are athletes, whose microbiome has relatively unique characteristics due to their long-term exercise. In addition, the way and intensity of a single exercise will also have different effects on gut microbiota (Donati Zeppa et al., 2019). At the same time, compared with long-term exercise intervention, there are relatively few studies on the changes of gut microbiota after one-time exercise, especially for non-athletes. However, some scholars believe that for exploring the acute changes of gut microbiota and metabolites caused by exercise, it is more conducive to clarify the mechanism of exercise affecting gut microbiota (Hughes and Holscher, 2021).

## 3 Effects of gut microbiota on exercise quality

Some studies have shown that the different basal state of gut microbiota may differentiate the training effect due to the different basal state of gut microbiota in the test groups. Therefore, the authors believe that the gut microbiota in the basal state can predict the body’s response to exercise training (Dupuit et al., 2022). This also indirectly indicates that gut microbiota can affect the body’s exercise ability. At present, the research focuses on the relationship between gut microbiota and strength and endurance quality (see Table 1).

**Table 1:** Effects of exercise on gut microbiota.

Mode of exercise		Participants	Changes in the diversity of gut microbiota
Long term exercise	Rugby (Campbell and Wisniewski, 2017)	Rugby players, non-players	Rugby players had a higher diversity of gut microbiota than non-athletes of similar age and BMI, and the higher the level of rugby players, the higher the relative expression of *Ruminococcaceae*, *Succinivibrionaceae*, *Succinivibrio* and *Akkermansiaceae* in the gut microbiota
	Cycling (Petersen et al., 2017)	Cyclist	Cyclists who exercised more than 16 h weekly had higher abundance of *Prevotella*.
	Cross-country sports (Fart et al., 2020)	Elderly athletes, ordinary elderly people	Older athletes have more beneficial bacteria and fewer harmful bacteria in their gut.
	High-intensity interval training plus resistance training three times a week for 12 weeks (Dupuit et al., 2022)	Overweight or obese postmenopausal women	The composition of gut microbiota changed: some microbiota were associated with changes in body composition. For example, the abundance of *Bifidobacterium* was positively correlated with fat mass and negatively correlated with muscle mass. However, the relationship between *Prevotella* and fat mass and muscle mass was negatively correlated.
	Undertake 30–60 min of endurance exercise three times a week for 6 weeks, followed by a return to sedentary behavior for 6 weeks (Allen et al., 2018)	Sedentary adults (BMI < 25)	1. *Faecalibacterium* and *Larneria* in gut microbiota increased significantly, while *Collinella* index decreased slightly after exercise intervention; 2. After 6 weeks of sedentary behavior, the changes of the above microbiota were reversed, especially in lean subjects; 3. Fecal SCFA produced by gut microbiota tended to increase, and acetate content continued to increase during the 6-week recovery period after exercise, while propionate and butyrate content decreased toward baseline.
		Sedentary adults (BMI > 30)	1. The indexes of *Collinella* and *Larsiella* increased, while the indexes of *Faecalibacterium* decreased significantly. 2. Six weeks after returning to sedentary behavior, all the above microbiota had reversed changes. 3. There was also an increase in SCFA, but the change was not significant, and the ability of gut microbiota to produce SCFA decreased during the 6-week recovery period.
	Wuqinxi exercise intervention of 40–50 min a day, 6 days a week for 6 months (Sun and Zhong, 2019)	Patients with Metabolic Syndrome	The intestinal microecology of the host was optimized. *Bifidobacterium*, *Lactobacillus*, *Bacteroides* and *Clostridium* in the patients increased, while *Clostridium*, *Enterococcus*, *Staphylococcus* and *Veillonella* decreased. And the longer the intervention time, the more obvious the effect.
One time exer-cise	Marathon race (Makin, 2021)	Marathon runner	The abundance of *Veillonella* was increased in athletes.
	Thirty-three consecutive days, 5,000 km ocean boat race (Keohane et al., 2019)	Four male athletes	The alpha diversity of the gut microbiota and the overall relative abundance of certain microorganisms in athletes have increased, manifested by the expression of gut microbiota genes involved in the biosynthesis of specific amino acids and fatty acids (such as *B. vulgatus* and *F. prausnitzii*, which are involved in S-adenosylmethionine synthesis), as well as the expression of *Coprococcus sp*. *ART55/1*, *R. intestinalis*, *B. vulgatus*, and *B. uniformis*, which are involved in the L-isoleucine metabolism pathway.
	Single moderate intensity exhaustion exercise (Tabone et al., 2021)	Male non-professional cross-country runner	1. There was no significant change in the composition diversity of gut microbiota. 2. The abundance of some gut microbiota and amino acid metabolites (such as tryptophan, tyrosine, and phenylalanine) were increased. 3. Metabolomics analysis found that some metabolites in serum came from gut microbiota.

### 3.1 Gut microbiota and strength quality

Six-week-old male ICR mice were gavaged with different doses of *Lactiplantibacillus plantarum* PL-02 (without PL-02, one dose, two doses and five doses) for 4 weeks. It was found that the average forelimb grip strength of the four groups of mice increased with the increase of PL-02 dose, and the PL-02 gavage group was significantly higher than the control group (Lee et al., 2021). Gut microbiota can also delay the weakening process of grip strength caused by aging in rats (Park et al., 2021). Mice that received gut microbiota from more physically fit older adults had higher grip strength (Fielding et al., 2019). Furthermore, in patients with end-stage renal disease, those with higher abundance of butyric acid-producing bacteria had stronger grip strength, thicker upper arms, and higher body mass index (Hu et al., 2022).

The promoting effect of gut microbiota on strength quality may be related to the promotion of skeletal muscle hypertrophy by gut microbiota. For example, 14-week-old C57BL/6 mice were divided into natural inoculation group (NAT), antibiotic treatment group (ATB) and control group (CTL). The CTL group was not treated, the ATB group was treated with oral antibiotics for 21 days, and the NAT group was treated with oral antibiotics for the first 10 days, followed by inoculation with gut microbiota for the next 11 days to remove the influence of antibiotics. It was found that the weight index of the extensor digital muscle and soleus muscle in the ATB group was not different from the other two groups, but the wet weight and weight index of the gastrocnemius muscle and quadriceps muscle were significantly lower than those of the other two groups (Nay et al., 2019). After 4 weeks of gavage of *Lactiplantibacillus plantarum* PL-02 selected from the intestine of athletes, it was found that the skeletal muscle mass of mice increased, and increased with the increase of *Lactiplantibacillus* dose (Lee et al., 2021).

Therefore, it is speculated that some kinds of gut microbiota may have a positive effect on increasing muscle strength, and this improvement of strength may be related to the increase of skeletal muscle mass promoted by the microbiota mentioned above, but there may also be other ways of action, which need further demonstration.

### 3.2 Gut microbiota and endurance quality

In the study of C57BL/6 mice, the endurance and endurance recovery ability of the ATB group were significantly lower than those of the NAT group, and the muscle fatigue index (defined as the time required to reduce the muscle output power to 50% of the maximum power by *in vitro* experiments) was also significantly lower than that of the NAT group and the control group (Nay et al., 2019), and this indicates that the impact of gut microbiota on endurance is dose-dependent (Lee et al., 2021). Some scholars believe that the improved endurance quality caused by the change of gut microbiota may be related to the metabolism of lactic acid. For example, studies have shown that *Veillonella* in the feces of athletes were isolated and injected into the gut of rats, and it was found that the exhaustion time of rats was longer. The authors believe that this change is because *Veillonella* can effectively metabolize lactate into propionate, thereby enhancing Cori cycle (Scheiman et al., 2019). However, some scholars believe that this result is not reliable, as the study’s use of mice fed with *Lactobacillus bulgaricus* as a control group is a confounding factor, as feeding with *Lactobacillus bulgaricus* is negatively correlated with endurance performance. Therefore, the author also believes that additional research is needed to confirm the relationship between microorganism administration, gut microbiota composition, and performance (Fernández-Sanjurjo et al., 2020).

Endurance quality is also affected by the body’s oxygen supply capacity and skeletal muscle energy material reserves. Studies have shown that the alpha diversity of gut microbiota is significantly correlated with maximal oxygen uptake (VO_2_ max) in sedentary healthy men after 150 min of moderate-intensity aerobic exercise per week compared with the control group without intervention (Resende et al., 2021). A similar conclusion has been obtained in studies on premenopausal women, that gut microbiota is correlated with cardiopulmonary function (Yang et al., 2017). In addition, exogenous supplementation of probiotics *Lactococcus lactis* sub sp. LY-66 and *Lactobacillus plantarum* PL-02 increased the maximum oxygen uptake of non-athletes (Lee et al., 2024).

In addition, the species and abundance of butyrate-producing microorganisms are positively correlated with cardiopulmonary fitness, and the increase in maximum oxygen uptake is simultaneous with the increase in the ratio of *Firmicutes* to *Bacteroidetes* (Mailing et al., 2019). It is suggested that gut microbiota may affect the ability of oxygen inhalation, transport and utilization. In addition, the production of bacterial-derived metabolites may affect the storage and availability of major energy substances in endurance exercise, such as glycogen and triglycerides, and affect the contractile function of skeletal muscle (Mancin et al., 2021), thus also affecting the endurance quality.

However, not all gut microbiota have a positive effect on the regulation of endurance quality, for example, the increase in the relative abundance of *Lactobacillaceae* will cause the weakening of endurance performance of C57BL/6 N mice (Fernández-Sanjurjo et al., 2020), and the composition of gut microbiota will not affect muscle fiber types or mitochondrial metabolism (Nay et al., 2019).

Based on the existing studies, it is found that the influence of gut microbiota on exercise quality is relatively concentrated. In addition to strength and endurance, there are few studies on other qualities (such as speed and agility). This may be because strength and endurance are the basic sports qualities, and their evaluation methods are relatively mature and well recognized. At present, the understanding of the reasons why gut microbiota affects exercise quality is not comprehensive, and its research mainly focuses on muscle status, cardiopulmonary function and other aspects. According to existing research results, it is also evident that physical fitness changes may only be related to specific gut microbiota, but whether other gut microbiota play a similar role still needs further research. In addition, the influence of gut microbiota on exercise may not only exist in the level of exercise quality, but also may be related to fatigue recovery and injury repair. Studies have found that oral administration of *Lactobacillus plantarum* PS128 is beneficial to reduce the damage of some bodies and organs of athletes caused by half marathon (Fu et al., 2021). Therefore, it is necessary to strengthen the research on gut microbiota and exercise quality in the future, and improve the cognition of the relationship between gut microbiota function and exercise.

## 4 Discussion on the mechanism of interaction between exercise and gut microbiota

### 4.1 To explore the mechanism of the effect of exercise on gut microbiota

At present, the mechanism of exercise on gut microbiota is not fully understood, and current research focuses on the following aspects.

#### 4.1.1 Exercise affects the gut microbiota by stimulating the gut

Gut microbiota is located in the intestinal lumen and can be affected by intestinal mucosal epithelial cells. Studies have found that the destruction of autophagy in intestinal epithelial cells can significantly change the composition of gut microbiota and reduce the alpha diversity of gut microbiota in mice (Soderholm and Pedicord, 2019). Exercise reduces the blood flow of intestinal organs and causes temporary hypoxia of intestinal epithelial cells with almost no difference in load intensity (Wu et al., 2020). Hypoxia activates autophagy (Li et al., 2022). In addition, specific knockout of Toll-like receptor4 (TLR4) intestinal epithelial cells can significantly affect the composition and function of gut microbiota related to lipid, amino acid and nucleotide metabolism (Soderholm and Pedicord, 2019). Aerobic exercise significantly reduces intestinal TLR4 in diabetic rats (Li et al., 2022), so it is speculated that exercise causes changes in gut microbiota through the above pathways.

Studies have also shown that temperature changes can cause changes in the diversity and composition of gut microbiota (Huus and Ley, 2021; Liu et al., 2023). For example, it has been shown that the *β*-diversity and composition of gut microbiota in free-wheeling mice changed significantly after exercise compared with non-exercise group. But this change did not occur at lower temperatures. Therefore, the authors suggest that exercise affects the composition of the gut microbiota by affecting core body temperature, including intestinal temperature (Sasaki et al., 2022). Some scholars believe that the influence of body temperature on gut microbiota may also be related to changes in metabolism, appetite and immune system function caused by temperature change (Huus and Ley, 2021), but the specific mechanism needs to be further studied. In addition, direct mechanical stimulation caused by exercise may be responsible for influencing the gut microbiota. For example, some studies suggest that exercise can enhance abdominal mechanical strength or promote the rapid passage of food through the colon by promoting the release of gastrointestinal hormones, thereby changing the PH value of the intestine and ultimately affecting the gut microbiota (Zhao et al., 2021).

#### 4.1.2 Exercise affects the gut microbiota via the immune system

The immune system may be an important mediator of the effects of exercise on gut microbiota. Both innate and adaptive immunity in the gut can affect the diversity and composition of gut microbiota (Yoo et al., 2020). Studies have shown that 2 weeks of moderate-intensity exercise and intermittent sprint exercise can effectively reduce circulating inflammatory factors and intestinal inflammatory markers, and increase the abundance of *Bacteroidetes* in people with insulin resistance. Reduce the ratio of *Firmicutes/Bacteroidetes* (Motiani et al., 2020). At the same time, due to the possible symbiotic relationship between immune cells and gut microbiota, the number and function of immune cells also affect the expression of gut microbiota. For example, studies have found that if Natural Killer T (NKT) cells are lacking, the pro-inflammatory microbiota will be increased (Selvanantham et al., 2016), but mice that are only reduced in invariant NKT (iNKT) will have increased anti-inflammatory microbiota and improved intestinal inflammation (Shen et al., 2018). In addition, some studies have shown that this condition can also affect the number of circulating immune cells [such as increased CD16^+^CD56^+^ NK cells and decreased CD3^+^ cytotoxic T cells (Tai et al., 2018)] and the proliferation ability of T cells (Navarro et al., 2013). It was also found that the number of lymphocytes, memory helper T (Th) cells, naive, memory, and activated cytotoxic T (Tc) cells, natural killer (NK) cells, NKT cells, and B1 cells were significantly reduced in peripheral blood samples of marathon runners compared to healthy sedentary controls (Panagoulias et al., 2023). These studies suggest that exercise may affect the function of gut or circulating immune cells, which in turn may affect the gut microbiota.

#### 4.1.3 Exercise affects gut microbiota via gut-brain axis

There is a brain-gut regulatory axis in the body, which is a bidirectional regulatory pathway, which is crucial for maintaining the homeostasis of the central nervous system and gastrointestinal tract. The brain can affect gut microbiota through the nervous system (sympathetic, parasympathetic) and endocrine (such as HPA axis, the hypothalamic–pituitary–adrenal axis) pathways (Hanscom et al., 2021). For example, studies have found that the use of drugs that reduce sympathetic nervous system excitability can attenuate the effects of exercise on gut microbiota (Sasaki et al., 2022). In addition, studies have shown that some patients with mental disorders have abnormal HPA axis function (most of them are hyperactive), and the composition of gut microbiota is different from that of healthy people. Therefore, some scholars believe that the nervous system can affect the gut microbiota through this endocrine pathway. At present, there are few direct studies on the relationship between exercise, HPA axis and gut microbiota. However, previous studies have shown that the activity of HPA axis is enhanced during exercise, but the negative feedback regulation of glucocorticoids will weaken the activity of HPA axis after exercise (Caplin et al., 2021). This may improve the disorder of gut microbiota caused by spirit, stress and other factors (Xie et al., 2022). But the results need further validation.

#### 4.1.4 Exercise affects gut microbiota via muscle factors

In addition to affecting gut microbiota through other systems, muscle contraction during exercise may also directly or indirectly cause changes in gut microbiota, and some muscle factors may play a role in this process. Irisin is a muscle factor secreted by exercise (Bilski et al., 2020), and it has also been found that exogenous irisin can effectively reverse the changes of gut microbiota caused by enteritis in mice (Huangfu et al., 2021). Therefore, irisin may mediate the changes in gut microbiota caused by exercise in diseases such as enteritis. TITIN, also known as C1q tumor necrosis factor-related protein 15 (CTRP15), is a newly discovered muscle factor that is released from rodent and human skeletal muscle in an exercise-induced manner (Tan et al., 2022; Shira et al., 2024). TITIN contributes to glucose and lipid metabolism; in addition, TITIN is positively correlated with IL-6 and TNF-*α*, suggesting its possible involvement in the inflammatory process (Shokoohi Nahrkhalaji et al., 2022). Among them, excessive glucose and fat intake can lead to the imbalance of gut microbiota (Zhang, 2022; Zeng et al., 2024). IL-6 deficiency can cause gut microbiota imbalance in mice (Wu et al., 2022). In a study of adolescents with depression, the content of *Bifidobacterium* in the gut microbiota of subjects was negatively correlated with the level of TNF-*α* (Chen X. et al., 2024). Therefore, it is speculated that TITIN may affect gut microbiota through some way. However, although some studies have found that some training modes (such as high-intensity interval training) can cause an increase in TITIN levels, not all training modes can cause changes in TITIN levels, which are related to the physiological conditions of the subjects themselves (Petro et al., 2024; Özçatal et al., 2023).

At present, there is little direct evidence that muscle factors affect gut microbiota, and the mechanism of action of muscle factors on gut microbiota needs to be further studied.

**Figure 1:**
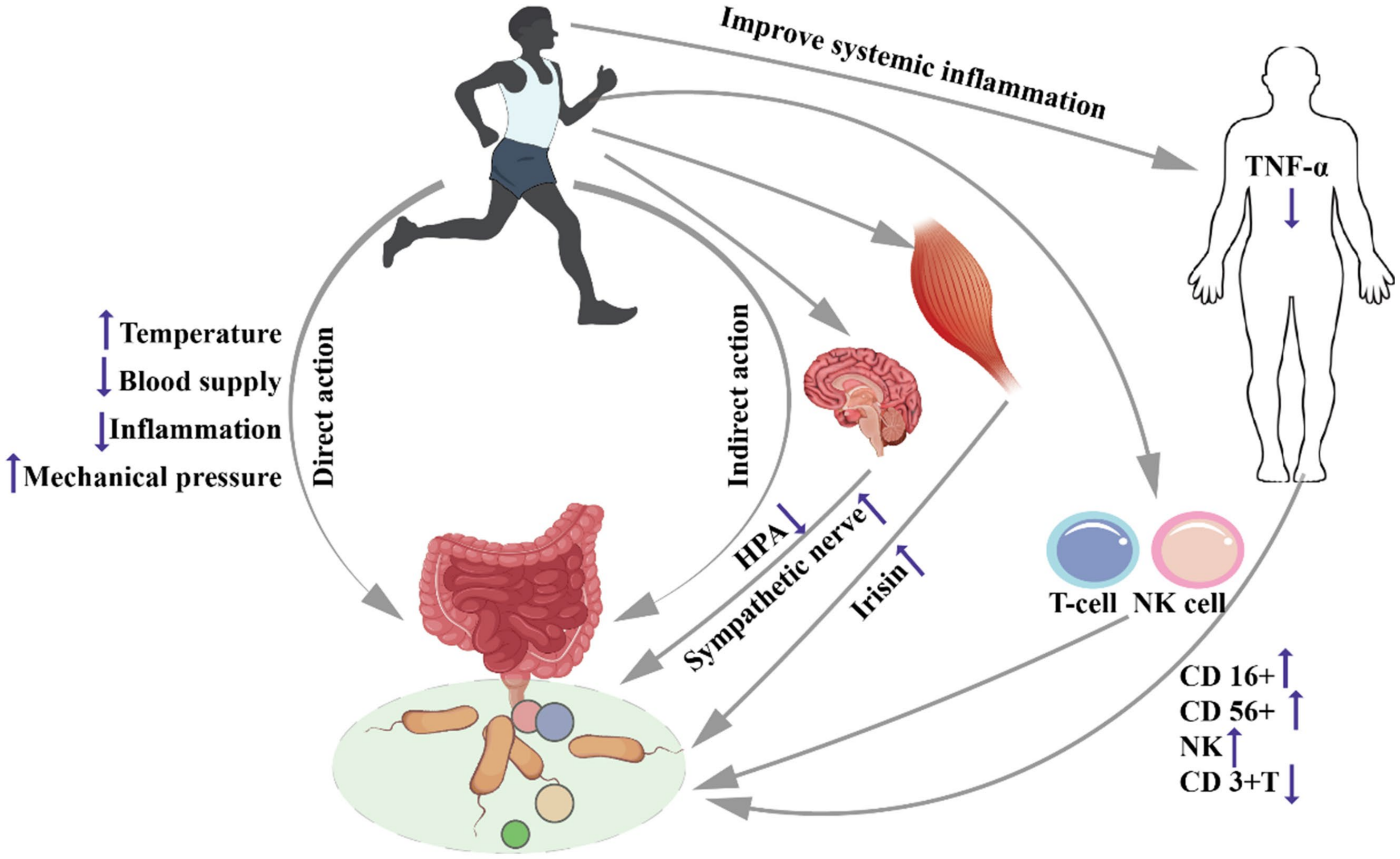
Mechanisms underlying the effects of exercise on gut microbiota. Effects of exercise on the intestine (1) Direct pathway: exercise induces changes in gut microbiota through changes in intestinal temperature, blood supply, inflammation and mechanical stress stimulation; (2) Indirect pathway: exercise affects intestinal flora by activating the central nervous system, promoting the release of muscle factors, changing the systemic and local inflammatory state, and the number of immune cells. Remarks: HPA: hypothalamic–pituitary–adrenal axis, T-cell: T lymphocyte, NK cells: natural killer cells.

In conclusion, there are two types of mechanisms (multiple pathways) of direct and indirect effects of exercise on gut microbiota, but the two are not independent (as shown in Figure 1). However, the specific action process of each mechanism has not been fully elucidated. Whether different exercise loads have different effects on different mechanisms needs to be further studied.

### 4.2 To explore the mechanism of the effect of gut microbiota on exercise capacity

#### 4.2.1 Gut microbiota may affect exercise capacity by changing muscle morphology and function

At present, there are relatively few studies on the mechanism of the direct effect of gut microbiota on exercise, and most of them are on the effect of gut microbiota on muscle. Studies have found that the effect of gut microbiota on muscle may be achieved by affecting protein and amino acid metabolism. For example, Lahiri et al. found that germ-free mice had lower skeletal muscle weight, fewer muscle fibers, higher expression of genes regulating skeletal muscle-related protein degradation, and lower expression of genes regulating skeletal muscle differentiation by comparing various indicators of germ-free and pathogen-free mice (Lahiri et al., 2019). Qiu et al. (2021) found that after administering antibiotics orally for 4 weeks, the gut microbiota of C57BL/6 mice was inhibited, and skeletal muscle atrophy was observed, accompanied by disturbances in bile acid metabolism in the small intestine, decreased farnesoid X receptor (FXR) and fibroblast growth factor 15 (FGF15) in the ileum, decreased circulating FGF15, and decreased levels of ERK and its phosphorylation in skeletal muscle; In addition, it was found that supplementation with FGF19 partially reversed skeletal muscle atrophy. Therefore, the authors suggest that gut microbiota may partly regulate ERK protein in skeletal muscle through the FXR-FGF15/19 signaling pathway in the ileum, thereby affecting protein synthesis in skeletal muscle. In addition, we found that circulating and hepatic FGF21 gene expression in normal was increased after restricted protein diet. However, when germ-free mice were fed a restricted protein diet, circulating and hepatic FGF21 gene expression did not change. Therefore, it can be seen that microorganisms use FGF21 to cope with changes in protein content in food to promote the synthesis of essential amino acids for the host to use, thereby inhibiting sarcopenia. Therefore, it can be seen that microorganisms use FGF21 to cope with changes in protein content in food to promote the synthesis of essential amino acids for the host to use, thereby inhibiting sarcopenia (Martin et al., 2021). Other studies have suggested that gut microbiota can not only improve muscle endurance and strength by affecting glycogen storage and utilization by skeletal muscle, but also enhance mitochondrial respiration by affecting IGF-1 content, thereby improving skeletal muscle mass (Zhao et al., 2021). The gut microbiota can also influence muscle status through its metabolites. As one of the main metabolites of gut microbiota, short-chain fatty acids can be absorbed by the intestinal lumen and regulate the metabolism and function of skeletal muscle (Frampton et al., 2020). After feeding sterile mice aged 6–8 weeks with water containing short chain fatty acids, a metabolic product of gut microbiota, for 4 weeks, the skeletal muscle dimension significantly increased and muscle strength was improved. This may be due to a decrease in the expression of the Atrogin-1 gene that causes muscle atrophy, while an increase in the expression of the MyoD gene that reflects muscle formation (Lahiri et al., 2019). Similar results have been obtained in human experiments. In the body of sarcopenia patients, the content of gut microbiota (such as *Faecalibacterium prausnitzii*) with the ability to metabolze SCFA is significantly reduced (Prokopidis et al., 2021). In menopausal women, metagenomic association analysis has found that the ability of intestinal microorganisms to synthesize SCFA butyrate is increased, which is significantly correlated with serum butyrate level and skeletal muscle index, and there is a causal relationship between intestinal butyrate synthesis and limb lean mass (Lv et al., 2021). A survey of 412 children found that the more SCFA in their feces, the greater their lean body mass and muscle mass in their limbs (Chen et al., 2022).

The reason why SCFA promote skeletal muscle synthesis and metabolism may be related to reducing the absorption of intestinal inflammatory substances and improving the inflammatory state of the body; In addition, it may also be related to the G protein receptor pathway promoting the secretion of glucagon like peptide-1 and casein, increasing insulin sensitivity, promoting mitochondrial synthesis, increasing type I muscle fiber composition, and promoting myoglobin expression (Prokopidis et al., 2021). In addition, some scholars have found that the relationship between SCFA and muscle mass in children is influenced by the body fat content of the subjects (Chen et al., 2022), suggesting that there may be an indirect pathway between gut microbiota metabolites and skeletal muscle mass. Based on the above research, it can be seen that the impact of gut microbiota on skeletal muscle quality is closely related to short chain fatty acids. However, further research is needed to investigate the effects and mechanisms of SCFA on gut microbiota, as well as whether there are age differences.

#### 4.2.2 Gut microbiota may influence exercise capacity through the nervous system

Studies suggest that there is a gut-brain regulatory axis in the body, and gut microbiota plays an important role in this regulatory axis. Some scholars have proposed the “microbiota-gut-brain” axis. Studies have shown that gut microbiota can affect the development and function of the nervous system from multiple ways (Cryan et al., 2019). Current studies have found that the influence of gut microbiota on motor behavior may be achieved by affecting the function of the nervous system. For example, some studies have shown that flies in the sterile state show hyperactive behavior, which is characterized by accelerated movement speed and increased daily activity, while the transplantation of specific microbiota has the opposite change, and this effect is involved in the neurons in the upper and lower esophageal regions of Drosophila (Schretter et al., 2018).

Gut microbiota through the nervous system may not only affect exercise behavior but also affect cardiopulmonary function. For example, in the study by Vicentini et al. (2021) it was found that after antibiotic treatment of adult mice, the neurons or glial cells in the small intestine were lost or reduced. After restoring the gut microbiota, the number of neurons and glial cells increased. In addition, studies have shown that gut microbiota can regulate microglia homeostasis and promote their death. Metabolites of gut microbiota, such as isoamyl amine (IAA), can induce apoptosis of microglia by activating the S100 calcium binding protein A8 (S100A8) signaling pathway (Loh et al., 2024). At the same time, some studies have shown that glutamate released by microglia is an important excitatory neurotransmitter that controls the heart and lung centers. In the study by Zoccal et al, glutamate injection was able to increase sympathetic nerve activity in the abdomen and chest of mice exposed to chronic intermittent hypoxia for 10 days and enhance cardiopulmonary function (Cohen et al., 2018). Therefore, gut microbiota can affect exercise capacity by changing the number and function of nerve cells.

In addition to this indirect approach, gut microbiota can also affect the levels of certain neurotransmitters, such as serotonin (5-HT) and gamma aminobutyric acid (GABA), which in turn affect the function of the nervous system (Cryan et al., 2019). Research has shown that drugs that increase the concentration of extracellular 5-HT often reduce the excitability of the human motor cortex and enhance the excitability of spinal motor neurons (Thorstensen et al., 2024). The regulation of GABA levels can enhance the recruitment of the sensory motor cortex. In addition, lower GABA levels are associated with poorer motor performance in older adults (Maes et al., 2022). However, the effect of gut microbiota on exercise capacity through this pathway needs to be further demonstrated.

At present, the mechanism by which gut microbiota affects exercise capacity through the nervous system is not fully understood. However, given the controlling and coordinating role of the nervous system in body function, the influence of gut microbiota on motor ability through the nervous system will be a promising research field.

#### 4.2.3 Other ways

In addition to muscle and nervous system pathways, gut microbiota also has an impact on exercise capacity through immune and metabolic pathways. Research has found that *Lactobacillus rhamnosus* can regulate the proliferation of intestinal T lymphocytes, thereby modulating host immune function (Shonyela et al., 2020). Supplementing probiotics can regulate serum cytokines and secreted immunoglobulin A, as well as the quantity and activity of innate and adaptive immune cells, and reduce post exercise inflammatory responses (Donati Zeppa et al., 2019). In addition, gut microbiota can influence exercise capacity through metabolic pathways. For example, research has found that supplementing with appropriate probiotics can improve the efficiency of energy supply in the body, play a role in promoting glucose uptake in skeletal muscle cells, increase gut microbiota-produced SCFA (such as acetate), and promote ATP production in skeletal muscle cells (Chen et al., 2022), delaying the occurrence of exercise-induced fatigue (Donati Zeppa et al., 2019). In the study by *Cheng et al.*, it was found that *Lactiplantibacillus plantum* TWK10 could reduce lactate production by promoting oxidation of fatty acids, and the administration of heat-inactivated TWK10 for 6 weeks showed potential benefits in anti-fatigue by reducing lactate and ammonia production during exercise (Cheng et al., 2023). The metabolic kinetic pathway of lactate labeled with isotope 13C3 showed that lactate accumulated in the blood after exercise could be transported to the intestine and metabolized to propionic acid by *Veillonella*, delaying fatigue and improving exercise capacity (Scheiman et al., 2019).

**Figure 2:**
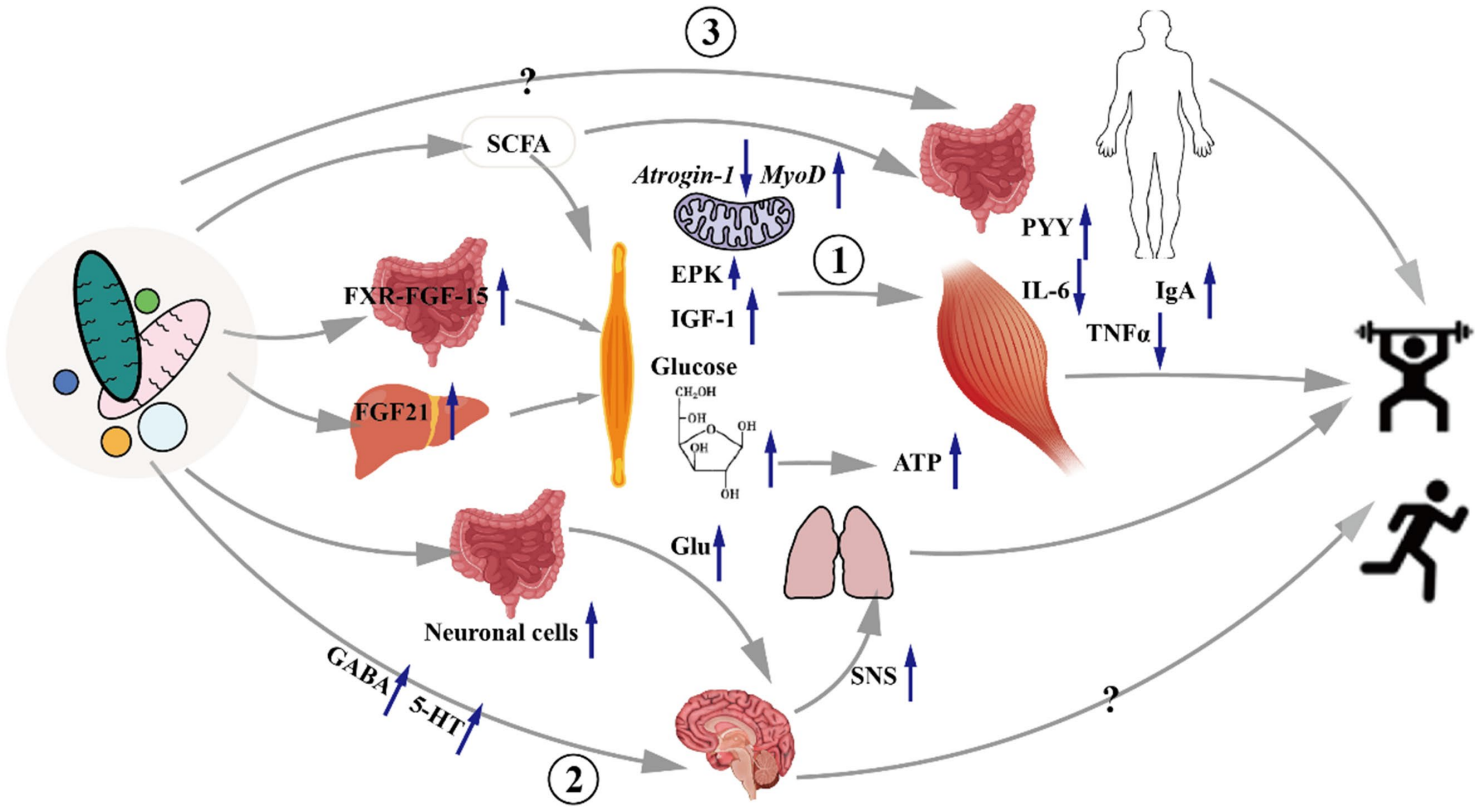
Mechanisms underlying the effects of gut microbiota on exercise capacity. At present, it is believed that the mechanism by which gut microbiota affects exercise capacity is mainly achieved by acting on skeletal muscle, nervous system, digestive system, immune system and other organ systems. The gut microbiota can promote the release of FGF21 from the liver through ileal bile acid-FXR-FGF15, and the metabolic products of the microbiota, SCFA, promote muscle protein synthesis, hypertrophy, and energy substance synthesis, which affect exercise ability; The intestinal tract can also synthesize neurotransmitters through the enteric nervous system to act on the central nervous system and affect the motor ability. Gut microbiota affects exercise capacity by changing the inflammatory state of the digestive system and the whole body and energy metabolism. Remarks: Glu: Glutamic acid, PYY: Peptide YY, SNS: Sympathetic nervous system.

There are few targeted studies on the effect of gut microbiota on exercise capacity, and the research results are mostly concentrated in the fields of disease and nutrition. The research content mainly focuses on muscle metabolism, inflammatory response, and nerve action (Figure 2). Therefore, the direct relationship and mechanism between gut microbiota and sports ability (especially competitive sports ability and sports quality) are not clear.

## 5 Conclusions and prospects

Exercise intervention can have an impact on the composition and structure of the gut microbiota, as well as the metabolites of the gut microbiota. However, this impact is not only related to the exercise intervention itself, but also to the basic state of the microbiota in the test population. Therefore, it is necessary to establish the effect of exercise on gut microbiota in different populations and strengthen the regular summary of the effect of long-term exercise intervention. The research on the mechanism of exercise affecting gut microbiota is still in the exploratory stage. Most of the research content is the observation of phenomena, and the effect of gut microbiota on skeletal muscle anabolism is more deeply studied, but the specific signaling pathways still need to be further clarified. Intervention with a single exercise session may be an effective means to study the mechanism of exercise affecting gut microbiota.

The intervention of gut microbiota may be an effective help to improve exercise capacity. The abundance, composition and diversity of gut microbiota can affect the body’s exercise quality. However, at present, the research on the effect of gut microbiota on exercise capacity is lack of pertinency, and the guiding and supporting role for exercise theory and practice is insufficient.
